# Maternal Supplementation With Krill Oil During Breastfeeding and Long-Chain Polyunsaturated Fatty Acids (LCPUFAs) Composition of Human Milk: A Feasibility Study

**DOI:** 10.3389/fped.2018.00407

**Published:** 2018-12-20

**Authors:** Anna Giulia Cimatti, Silvia Martini, Alessandra Munarini, Maximilano Zioutas, Francesca Vitali, Arianna Aceti, Vilma Mantovani, Giacomo Faldella, Luigi Corvaglia

**Affiliations:** ^1^Neonatal Intensive Care Unit, S. Orsola-Malpighi University Hospital, Bologna, Italy; ^2^Department of Medical and Surgical Sciences, University of Bologna, Bologna, Italy; ^3^Department of Clinical Medicine, Center of Applied Biochemical Research, S. Orsola-Malpighi University Hospital, Bologna, Italy; ^4^Pediatric Endocrinology, Department of Pediatrics, S. Orsola-Malpighi University Hospital, Bologna, Italy

**Keywords:** LCPUFA, DHA, AA, EPA, breast milk, supplementation, krill oil, lactation

## Abstract

**Background:** Docosahexaenoic acid (DHA) is a major constituent of neuronal and retinal membranes and plays a crucial role in brain and visual development within the first months of life. Dietary intakes are fundamental to provide neonates with adequate DHA supply; hence, maternal supplementation might represent a useful strategy to implement DHA contents in breast milk (BM), with possible benefits on neonatal neurodevelopment. *Antarctic krill* is a small crustacean rich in highly available phospholipid-bound DHA. This pilot study aimed to evaluate whether maternal supplementation with krill oil during breastfeeding increases long-chain polyunsaturated fatty acids (LCPUFAs) BM contents.

**Methods:** Mothers of infants admitted to the Neonatal Intensive Care Unit were enrolled in this open, randomized-controlled study between 4 and 6 weeks after delivery and randomly allocated in 2 groups. Group 1 received an oral krill oil-based supplement providing 250 mg/day of DHA and 70 mg/day of eicosapentaenoic acid (EPA) for 30 days; group 2 served as control. BM samples from both groups were collected at baseline (T0) and day 30 (T1) and underwent a qualitative analysis of LCPUFAs composition by gas chromatography/mass spectrometry.

**Results:** Sixteen breastfeeding women were included. Of these, 8 received krill-oil supplementation and 8 were randomized to the control group. Baseline percentage values of DHA (%DHA), arachidonic acid (%AA), and EPA (%EPA) did not differ between groups. A significant increase in %DHA (T0: median 0.23% [IQR 0.19;0.38], T1:0.42% [0.32;0.49], *p* 0.012) and %EPA (T0: median 0.10% [IQR 0.04;0.11], T1:0.11% [0.04;0.15], *p* 0.036) and a significant reduction in %AA (T0: median 0.48% [IQR 0.42;0.75], T1:0.43% [0.38;0.61], *p* 0.017) between T0 and T1 occurred in Group 1, whereas no difference was seen in Group 2. Consistently, a significant between-group difference was observed in percentage changes from baseline of DHA (Δ%DHA, group 1: median 64.2% [IQR 27.5;134.6], group 2: −7.8% [−12.1;−3.13], *p* 0.025) and EPA (Δ%EPA, group 1: median 39% [IQR 15.7;73.4]; group 2: −25.62% [−32.7;−3.4], *p* 0.035).

**Conclusions:** Oral krill oil supplementation effectively increases DHA and EPA contents in BM. Potential benefits of this strategy on brain and visual development in breastfed preterm neonates deserve further evaluation in targeted studies.

**Clinical Trial Registration**: www.ClinicalTrials.gov, identifier NCT03583502.

## Introduction

Long-chain polyunsaturated fatty acids (LCPUFAs), such as docosahexaenoic (DHA, 22:6 n−3) and arachidonic acid (AA, 20:4 n−6), are major building blocks for the lipid bilayer of neurons and retina. Brain maturation and visual development start during pregnancy and continue throughout the first year of life; consistently, LCPUFAs and, in particular, DHA, exert their greatest effect during this period (1, 2), and benefits on visual acuity and cognitive development have been largely established in term (3, 4) and preterm (5, 6) infants fed LCPUFAs-supplemented formula.

Like all mammals, humans lack enzymes for the synthesis of n−3 and n−6 precursors of DHA and AA, which are therefore essential fatty acids and need to be provided by dietary sources (7). Furthermore, consistently with the growth spurt of human brain occurring during the third trimester of pregnancy, placental transfer of AA and DHA is highest during this period (8); however, premature birth may preclude this LCPUFAs accretion, therefore dietary intakes are even more crucial in preterm neonates, who are at increased risk of neurodevelopmental impairment (9). In this delicate population, moreover, low blood DHA levels have been associated with a higher incidence of prematurity-related complications, such as intraventricular hemorrhage (IVH), bronchopulmonary dysplasia (BPD), and infections (10, 11).

Due to its several nutritional and non-nutritional benefits, breast milk (BM) is the optimal feeding choice for both term and preterm neonates (12, 13), and is generally regarded as providing adequate intakes of essential fatty acids. LCPUFAs contents of BM, however, are significantly influenced by maternal dietary intakes: as an example, fish-rich diets have been associated with high DHA levels in BM, whereas vegan dietary habits may lead to low concentrations of these compounds (14–16).

*Antarctic krill*, a small crustacean belonging to the order Euphausiacea, is by far the most dominant member of the Antarctic zooplankton community, and also represents a rich source of n-3 LCPUFAs (i.e., DHA and eicosapentaenoic acid [EPA]). Differently from other LCPUFAs sources, krill oil DHA and EPA are mainly bound to phosphatidylcholine, resulting in a significantly improved bioavailability (17). Hence, maternal krill oil supplementation might represent an effective strategy to enhance LCPUFAs contents in human milk, with possible beneficial effects on visual and brain development of breastfed neonates, especially if born preterm. To date, however, whether oral maternal supplementation with krill oil during breastfeeding increases BM concentration of LCPUFAs has not been investigated yet.

The aim of this pilot study was to evaluate the effect of a combined krill and fish oil oral supplementation, administered in breastfeeding women, on LCPUFAs composition of BM.

## Methods

### Study Population and Ethics

Breastfeeding mothers of infants admitted at the Neonatal Intensive Care Unit of Sant'Orsola-Malpighi University Hospital, Bologna, Italy, were consecutively enrolled between 4 and 6 weeks after delivery, when peak lactation was already established and breast milk can be considered fully mature (18), if a written informed consent to participate in the present study was obtained. Ongoing LCPUFAs supplementation at the time of enrollment was considered an exclusion criterion.

The study protocol was approved by the Ethics Committee of Sant'Orsola-Malpighi University Hospital, Bologna, Italy (Study ID: 114/2015/U/Sper) and is registered in the Protocol Registration System ClinicalTrials.gov (NCT03583502).

### Study Design

The lactating women enrolled in this open, randomized, controlled trial were randomly allocated to 2 groups. Group 1 received 2 gelatin soft capsules per day of a combined krill and fish oil supplement (Krilling D®, Italchimici S.P.A., Milan, Italy), providing 250 mg/day of DHA and 70 mg/day of EPA, for overall 30 days, whereas group 2 served as control. Approximately 10 ml of fresh mid-BM samples were collected from both groups at baseline (T0) and at day 30 (T1). In order to minimize the effect of dynamic changes in LCPUFAs excretion within the day (18, 19), the study samples were collected at early morning. After collection, the samples were stored at −80°C until ready for extraction to minimize lipid oxidation and degradation.

At T0, the enrolled women were also asked to fill out a food frequency questionnaire (FFQ, Supplementary Table 1), aimed at calculating individual dietary intakes of LCPUFAs based on their food habits over the last month. This FFQ investigated the consumption of 20 food types containing polyunsaturated fatty acids, including supplements. For each one, average portions (never consumed, less than once a week, 1/week, 2/week, 3/week, or more than 3/week) were reported. If the average portion consumption was >3 times a week, the patient was instructed to provide the exact number of portions actually consumed per week. Once the FFQ was completed, the average consumption of linoleic acid (LA), alpha-linolenic acid (ALA), AA, EPA, DHA, n-6, and n-3, expressed in grams per week, was calculated for each study subject. Based on population reference intakes in pregnancy and breastfeeding, according to the Italian recommended daily allowance (RDA) (20), the proportion of breastfeeding women who did not reach the recommended EPA and DHA intakes (EPA+DHA: 2.8 g/week) was calculated.

An intermediate evaluation (by visit or phone call) of group 1 compliance and adherence to the ongoing supplementation was performed at day 15.

### Gas Chromatography/Mass Spectrometry (GC-MS) Analysis

Qualitative analysis of BM LCPUFAs composition was performed at the laboratory of the Center for Applied Biomedical Research (CRBA) of Sant'Orsola-Malpighi University Hospital, Bologna, Italy, by means of GC-MS.

After bringing BM samples at room temperature under continuous mixing, 0.5 ml from each sample were transferred to Sovirel extraction tubes and extracted twice with chloroform/methanol (2:1 vol/vol, 3+2 ml) containing butylated hydroxytoluene 0.01% as anti-oxidant^10^.

After centrifugation (400xG, 10 min 25°C), the organic phases were combined, re-extracted with chloroform/H_2_O (1:1, vol/vol, 2+2 ml) and separated by centrifugation; the lowest organic phase was then transferred to a new tube and dried under nitrogen stream at 30°C.

The phospholipid fraction was saponified into free fatty acids via a base-catalyzed reaction (KOH 0.5 M, 2 ml in methanol) and esterified to fatty acid methyl esters (FAMEs) by acid reaction with boron trifluoride (14% in methanol), 1 ml for 10 min at 80°C.

After cooling, FAMEs were extracted twice into hexane (3+2 ml), dried under nitrogen stream and dissolved in cyclohexane for direct injection into a gas chromatograph (Agilent HP5890), equipped with a programmed temperature vaporizing (PTV) injector.

FAMEs were then separated and identified by a mass spectrometric detector (MSD, Agilent 5973, Agilent Technologies Cheadle, UK) in electron impact ionization mode (70 eV) using a Supelco SP^TM^ 2330 column (30 mt × 0.25 mm × 0.2 μm film thickness) with helium as carrier gas (initial flow 0.5 ml/min, constant pressure mode, 10.8 psi).

PTV injections were carried out in solvent vent mode; the initial temperature was set at 60°C, with a purge flow of 50 mL/min for 0.30 sec; the temperature was then ramped at 720°C/min to 220°C, and held for the whole analysis period.

The oven temperature was initially held at 100°C (cold-trapping technique) for 1.25 min, then ramped at rate of 30°C/min to 185°C, held 10 min, further increased at 5°C/min to 205°C and held 10 min (total run time: 28.08 min). The MSD ion source, quadrupole and transfer line temperatures were 150, 230, and 280°C, respectively. Total ion chromatography (40–550 m/z, 100 dwell time) was used for spectral percentage quantitation.

The analytes were identified by comparison with standards retention time (Supelco 37 Component FAME Mix, Sigma Aldrich), whereas mass spectral identity was confirmed by comparison with WileyN and NIST mass spectral databases. Peak areas calculations were performed using the Agilent G1701 BA software.

Within-batch and day-to-day reproducibility were calculated splitting a basal sample into 3 aliquots, which were independently analyzed on the same day; the obtained values are reported in Supplementary Table 2. Accuracy was tested vs. FAMEs certified standard material (Supelco, CRM 47885).

Percentage quantitation of each FA was expressed as its percentage over total fatty acid contents (i.e., palmitic, stearic, oleic, docosapentaenoic acid, LA, ALA, AA, EPA, DHA), calculated according to the following formula:

$$\begin{array}{l} Analyte\;percentage\left(\%\right)=\frac{Analyte\;Peak\;area\;*100}{\left(\sum \;peak\;areas\;of\;the\;identified\;analytes\right)} \end{array}$$

### Statistical Analysis

IBM SPSS Statistics (Statistical Package for the Social Sciences, SPSS Inc., Chicago, IL, USA) version 25 was used for statistical analysis. Intra-group differences in median percentage values of DHA, EPA and AA between T0 and T1 were evaluated by Wilcoxon signed-rank test. Mann-Whitney *U*-test was used to compare clinical characteristics, LCPUFAs dietary intakes and percentage changes from baseline of DHA, EPA, and AA between the two study groups. Fisher's exact test was used to compare the prevalence of inadequate DHA+EPA dietary intakes between the two groups. Eventually, a linear regression model was built to adjust the results for possible influencing factors. Significance level was set at *p* < 0.05.

## Results

**Figure 1:**
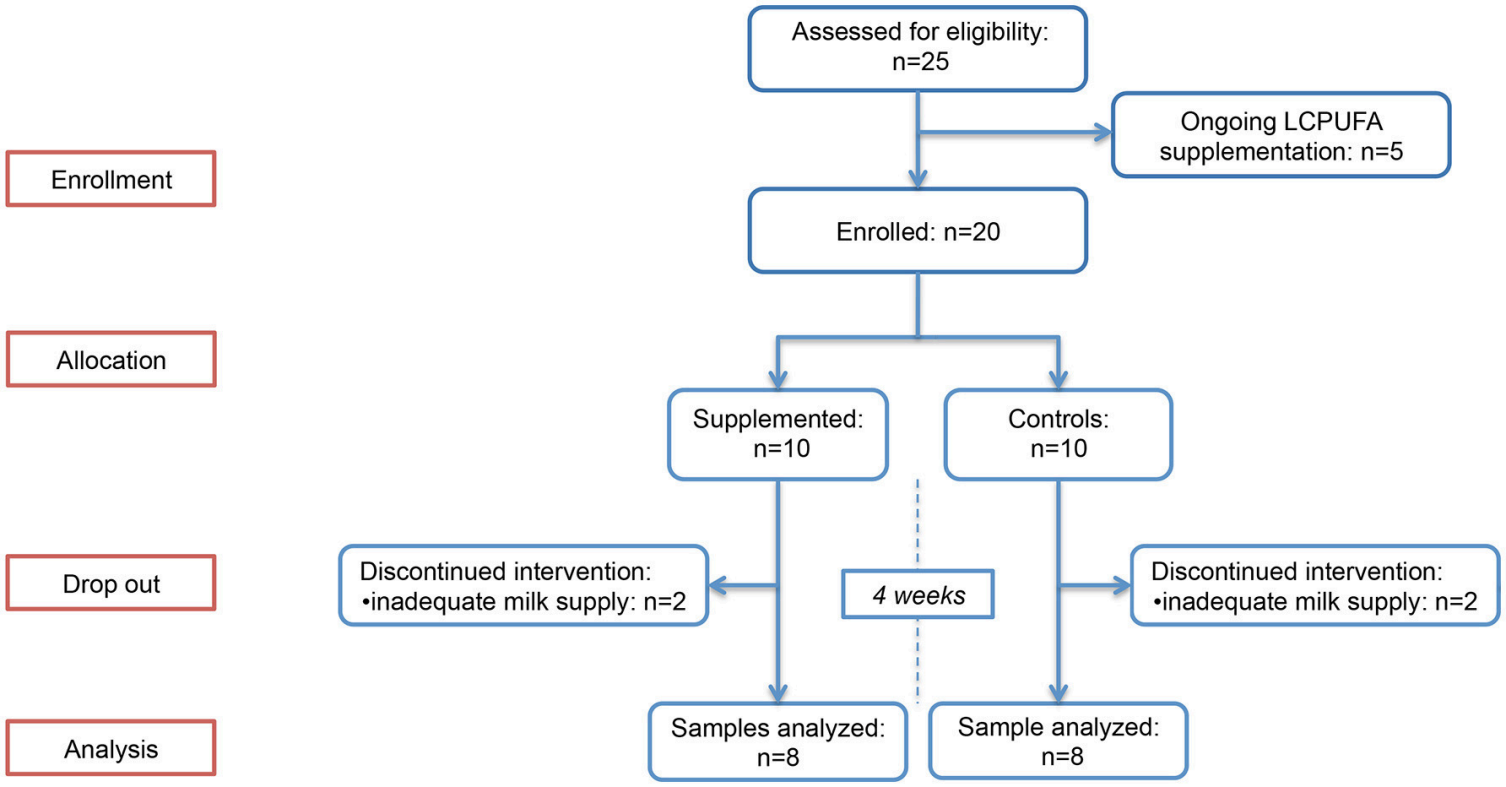
Flow diagram of the main study phases.

In total, twenty breastfeeding women were enrolled and randomly allocated in the two study groups. Of these, 4 (2 in group 1 and 2 in group 2) had a significant decrease in breast milk supply between T0 and T1, and were excluded from the study due to the unavailability of T1 sample; therefore, 8 women in group 1 and 8 in group 2 were included in the study. The flowchart summarizing the progress of women enrolled through the trial is provided in Figure 1. Baseline BM samples were collected at a median of 34 (interquartile range [IQR]: 31–38) days after delivery. No difference between the two groups was observed in LCPUFAs dietary intakes, estimated by FFQ and summarized in Table 1. The infants' characteristics are provided in Supplementary Table 3; no between-group difference in postconceptional age was observed at the time of sample collection (*p* = 0.798).

**Table 1:** Weekly dietary intakes of linoleic acid (LA), alpha-linolenic acid (ALA), arachidonic acid (AA), eicosapentaenoic acid (EPA), docosahexaenoic acid (DHA), ω-6 and ω-3 in the study groups, estimated by food-frequency questionnaire.

**Dietary intakes, median (interquartile range [IQR])**	**Group 1 (*n* = 8)**	**Group 2 (*n* = 8)**	**Group 1 vs. 2, *p*-value**
LA, g/sett	33.70 (24.82–48.25)	36.41 (32.20–44.41)	0.606
ALA, g/sett	3.15 (2.64–4.65)	3.39 (2.92–4.31)	0.797
AA, g/sett	0.94 (0.53–1.24)	1.04 (0.72–2.41)	0.298
EPA, g/sett	0.77 (0.65–1.55)	0.76 (0.50–2.28)	0.898
DHA, g/sett	1.42 (1.03–2.65)	1.12 (0.67–3.95)	1.000
ω-6, g/sett	34.78 (25.79–49.24)	37.30 (32.99–46.83)	0.606
ω-3, g/sett	5.67 (4.64–8.12)	5.53 (4.12–10.39)	1.000

Based on FFQ reports, 5 women in group 1 and 3 in group 2 (between-group difference *p* = 0.399) had lower DHA intakes than the recommended dietary reference. Of interest, DHA and EPA supplementation (250 and 75 mg/die, respectively) normalized DHA and EPA intakes in the 5 women, randomized to group 1, with inadequate dietary intakes.

Median percentage values of DHA (%DHA), AA (%AA), and EPA (%EPA) for the two study groups are detailed in Table 2. Baseline %DHA, %AA, and %EPA did not differ between groups. Group 1 showed a significant increase in %DHA and %EPA and a significant reduction of %AA between T0 and T1. Group 2 showed a decrease in %DHA, %EPA, and %AA between T0 and T1; statistical significance was observed for %AA reduction.

**Table 2:** Median percentage values of docosahexaenoic acid (DHA), arachidonic acid (AA) and eicosapentaenoic acid (EPA) for the two study groups and results of intra-group comparison between T0 and T1.

**Percentage values, median (interquartile range [IQR])**	**Group 1 (*****n*** **=** **8)**			**Group 2 (*****n*** **=** **8)**		
	**T0**	**T1**	**T0 vs. T1, *p*-value**	**T0**	**T1**	**T0 vs. T1, *p*-value**
DHA, %	0.23 (0.19–0.38)	0.42 (0.32–0.49)	0.012	0.38 (0.21–0.57)	0.28 (0.20–0.50)	0.208
EPA, %	0.10 (0.04–0.11)	0.11 (0.04–0.15)	0.036	0.14 (0.08–0.20)	0.10 (0.08–0.13)	0.093
AA, %	0.48 (0.42–0.75)	0.43 (0.38–0.61)	0.017	0.65 (0.52–0.95)	0.60 (0.44–0.74)	0.036

Consistently, a significant between-group difference was observed in percentage changes from baseline of DHA (Δ%DHA, group 1: median 64.2% [interquartile range, IQR: 27.5;134.6], group 2: −7.8% [−12.1; −3.13], p 0.025) and EPA (Δ%EPA, group 1: median 39% [IQR 15.7;73.4]; group 2: median −25.62% [−32.7;−3.4], *p* 0.035).

**Figure 2:**
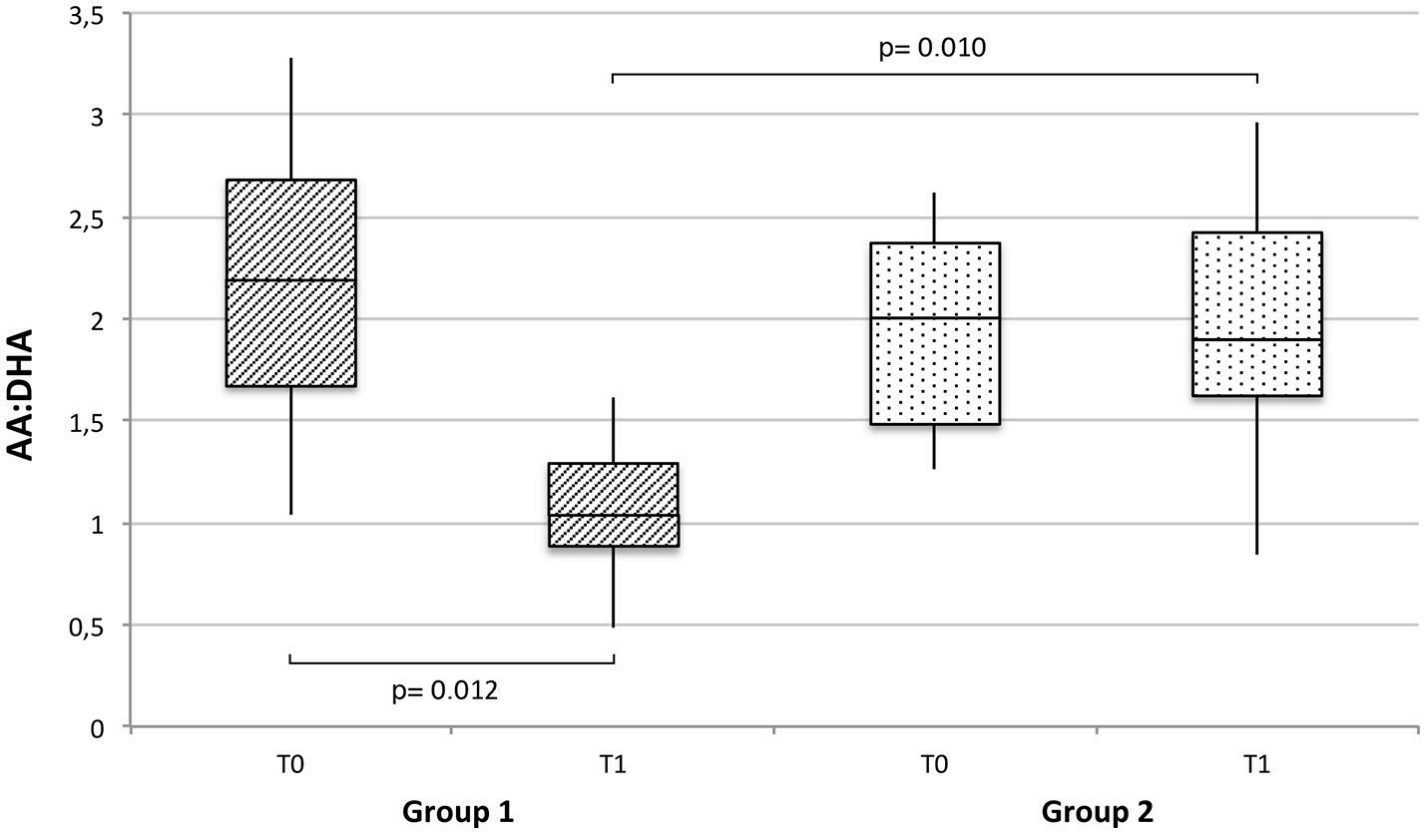
Arachidonic (AA) to docosahexaenoic acid (DHA) ratio (AA:DHA), expressed as median, interquartile range and range, at baseline and T1 in breast milk of supplemented (group 1) vs. non-supplemented (group 2) lactating women.

The ratio between AA and DHA (AA:DHA), which provides an estimation for n−6/n−3 fatty acid ratio, was also calculated for each study group at T0 and T1. As shown in Figure 2, a significant decrease (*p* = 0.012) in AA:DHA was observed in the supplemented group between T0 an T1, resulting in significantly lower AA:DHA values at T1 compared to the control group (*p* = 0.010).

Given the well-known influence of maternal dietary intakes on BM LCPUFA composition, a multivariate linear regression model was built to adjust Δ%DHA and Δ%EPA (dependent variables) for the respective dietary intakes calculated by the FFQ and baseline %DHA and %EPA concentrations (independent variables). The regression analysis, whose results are summarized in Table 3, confirmed a significant effect of DHA and EPA supplementation on the percentage observed.

**Table 3:** Linear regression model for percentage changes of docosahexaenoic acid (Δ%DHA) and eicosapentaenoic acid (Δ%EPA) from baseline.

**Dependent variable**	**Model**		**B**	**SE B**	**Beta**	***t***	***P***
Δ%DHA	R^2^0.495	Constant	2.331	55.345		0.042	0.967
		dietary DHA intake	-7.860	14.416	-0.135	-0.545	0.599
		baseline %DHA	-10.435	123.660	-0.022	-0.084	0.935
		supplementation	98.833	36.782	0.666	2.687	0.025
Δ%EPA	R^2^0.569	Constant	16.614	33.868		0.491	0.635
		dietary EPA intake	-8.108	18.968	-0.112	-0.427	0.679
		baseline %EPA	-246.928	265.832	-0.256	-0.929	0.377
		supplementation	60.321	24.395	0.579	2.473	0.035

## Discussion

According to the present results, a 30-day combined supplementation with fish and krill oil in breastfeeding women significantly increases BM DHA and EPA levels.

The supportive effects of n−3 fatty acids and, in particular, of DHA, on visual and cognitive development in early life have long been known. On the other hand, poor dietary intakes of DHA have been shown to decrease DHA contents in cerebral cortex and retinal membranes, with possible consequences on visual and cognitive functions (21), and low blood levels in preterm infants have been associated with increased rates of IVH (10), late-onset sepsis and BPD (11).

Human milk is considered the best nutritional choice for both term and preterm infants; BM LCPUFAs composition, however, reflects the nutritional status of the breastfeeding mother. Consistently, either 6 or 12-week maternal DHA supplementation effectively increased plasma DHA levels in breastfed infants compared to the placebo group (22, 23). According to a recent meta-analysis, maternal DHA supplementation during breastfeeding is associated with an improved neurodevelopmental pace up to 3 years of life in BM-fed preterm infants (24); moreover, term neonates whose mothers underwent DHA supplementation for the first 4 month of breastfeeding scored significantly better than controls on sustained attention scales at 5 years of age (25), further contributing to support the beneficial effects of DHA supplementation during breastfeeding.

DHA supplements (22, 26, 27) or fish oil administration (28) during lactation have proved to effectively modify LCPUFAs concentration not only in maternal plasma, but also in BM. However, to the best of our knowledge, this is the first study aimed at investigating the effects of a combined krill and fish oil supplementation on BM LCPUFAs composition. Differently from fish oils, whose EPA and DHA are predominantly bound to triglycerides, *Antarctic Krill* LCPUFAs are incorporated into phospholipids, which seem to be associated with enhanced bioavailability and better absorption (29).

According to our results, a 30-day combined krill and fish oil supplementation has led to a significant increase in BM %DHA and %EPA. On the other hand, a slight decrease in %DHA (−25%) and %EPA (−29%) was observed in the control group, whereas %AA was significantly reduced in both the study groups.

The latter finding is consistent with the progressive decrease in BM LCPUFAs for increasing lactation phases (30). Moreover, n−3 fatty acids such as DHA and EPA act as competitive substrates for the enzymes and products of arachidonic acid metabolism (31), and this may have further contributed to the significant %AA reduction in the supplemented group.

AA:DHA ratio has been used as a marker for DHA variations in biological samples (22), and its decrease may also reflect the biological effect of n−3 series, due to the above mentioned enzymatic competition between DHA and AA production (32). In neonatal rat models, changes in the dietary n−6/n−3 ratio significantly altered the fatty acid composition of neurons, glial membranes, and developing retinal photoreceptors, which reflected the administered dietary proportion (33, 34). In human preterm neonates, higher DHA and lower LA blood levels in the first few weeks of life, resulting in a decreased AA:DHA ratio, have been associated with improved microstructural brain development, reduced IVH incidence and better developmental scores at follow-up (10).

Placental transfer of DHA is greatest during the third trimester of pregnancy, consistently with the LCPUFAs-dependent growth spurt of human brain occurring during this period (35). In case of premature birth, this transfer is disrupted and inadequate dietary intakes of LCPUFAs may be detrimental. In this phase, particular attention should be paid to achieve and maintain appropriate DHA levels, as suggested by the evidence of improved brain development and better cognitive outcomes in preterm infants with higher DHA levels in red blood cells over the first weeks of life (10).

According to our preliminary results, combined krill and fish oil supplementation might represent an easy and feasible strategy to increase BM DHA levels not only in breastfeeding mothers of preterm neonates, but also in donors of Human Milk Banks, whose milk is often used for preterm infants' enteral nutrition when own mother's milk is lacking, but it is reported to contain significantly lower DHA levels than published values for maternal milk and infant formula and thus may not meet the recommended provision for this delicate population (36).

The following study limitations need to be acknowledged. First, sample size calculation was not performed due to the pilot nature of the study. However, a *post-hoc* calculation based on the observed %DHA values has shown that the actual power of the study is 66.5%, whereas 10 women per group would be adequate to achieve a power of 80%. Moreover, LCPUFAs concentration in maternal plasma, which may have provided additional information on the supplement absorption and excretion rate, was not investigated. Eventually, in this study supplement krill oil was combined to fish oil; hence, it is not possible to determine the exact impact of each of them in determining the observed increase in %DHA and %EPA.

Further studies are needed to confirm these preliminary findings, and to quantify krill oil absorption and excretion in breastfeeding women. Furthermore, potential benefits of krill oil supplementation, such as the increase in LCPUFA blood levels in preterm infants fed either maternal or donor milk, and the improvement of these infants' neurocognitive and visual outcomes, deserve to be assessed in targeted clinical trials.

## Author Contributions

LC, GF, AM, and VM designed the study. AA, FV, AC, and SM collected the data. AM carried out the laboratory analysis. MZ designed the food-frequency questionnaire and calculated dietary intakes. SM performed statistical analysis. AC and SM wrote the first draft. All the authors critically reviewed the manuscript and approved the final version submitted for publication.

### Conflict of Interest Statement

The authors declare that the research was conducted in the absence of any commercial or financial relationships that could be construed as a potential conflict of interest.

## Abbreviations

AA: arachidonic acid
ALA: alpha-linolenic acid
BM: breast milk
BPD: bronchopulmonary dysplasia
DHA: docosahexaenoic acid
EPA: eicosapentaenoic acid
FAMEs: fatty acid methyl esters
FFQ: food frequency questionnaire
IQR: interquartile range
IVH: intraventricular hemorrhage
LA: linoleic acid
LCPUFAs: long-chain polyunsaturated fatty acids
MSD: mass spectrometric detector
PTV: programmed temperature vaporizing
RDA: recommended daily allowance.
