# Supplementary material for: Maternal Supplementation With Krill Oil During Breastfeeding and Long-Chain Polyunsaturated Fatty Acids (LCPUFAs) Composition of Human Milk: A Feasibility Study
Source: Front Pediatr. 2018 Dec 20;6:407. doi: 10.3389/fped.2018.00407 (PMC6308297; doi:10.3389/fped.2018.00407)
Supplement: Supplementary file 1 [file Table_1.DOCX]

**Food frequency questionnaire for polyunsaturated fatty acids intakes**

Tick the boxes according to the consumption frequency over the last month for each food marked on the left column. If the food consumption is greater than 3 times a week, sign the number of portions normally consumed in the week in the column "> 3 times / week" (example: 2 tablespoons of olive oil a day are equivalent to 14 servings a week)

|  | >3/week | 3 /week | 2 /week | 1 /week | <1/week | Never |
| --- | --- | --- | --- | --- | --- | --- |
| **Walnuts** 1 portion 20 g – 3 walnuts |  |  |  |  |  |  |
| **Other dried fruit**  1 portion 20 g |  |  |  |  |  |  |
| **Blue fish**  (sardines, salmon, fresh tuna, anchovies, mackerel, herring)  1 portion 150 g |  |  |  |  |  |  |
| **Tuna in oil, drained**  1 portion 1 can of 52 g |  |  |  |  |  |  |
| **Other fish**  1 portion 150 g |  |  |  |  |  |  |
| **Molluscs and Crustaceans**  1 portion 150 g |  |  |  |  |  |  |
| **Seed oil**  1 portion 1 tablespoon |  |  |  |  |  |  |
| **Olive oil**  1 portion 1 tablespoon |  |  |  |  |  |  |
| **lard, mayonnaise**  1 portion 1 tablespoon |  |  |  |  |  |  |
| **Food rich in palm oil**  (snacks, shortbread biscuits, chips)  1 portion 30 g – 3 biscuits |  |  |  |  |  |  |
| **Egg (yolk)**  1 portion 1 egg |  |  |  |  |  |  |
| **Pasta, bread and rice**  1 portion 100 g |  |  |  |  |  |  |
| **Cereals** (oats, corn, barley, wheat, buckwheat)  1 portion 50 g |  |  |  |  |  |  |
| **Cheese**  1 portion 40 g |  |  |  |  |  |  |
| **Veal and beef**  1 portion 120 g |  |  |  |  |  |  |
| **Pork**  1 portion 120 g |  |  |  |  |  |  |
| **White meat**  1 portion 120 g |  |  |  |  |  |  |
| **Cured meat**  1 portion 40 g |  |  |  |  |  |  |
| **Stuffed desserts**  1 portion 100 g |  |  |  |  |  |  |
| **Omega 3 supplements**  1 portion 250 mg – 2 capsules |  |  |  |  |  |  |
