# Supplementary material for: Maternal Supplementation With Krill Oil During Breastfeeding and Long-Chain Polyunsaturated Fatty Acids (LCPUFAs) Composition of Human Milk: A Feasibility Study
Source: Front Pediatr. 2018 Dec 20;6:407. doi: 10.3389/fped.2018.00407 (PMC6308297; doi:10.3389/fped.2018.00407)
Supplement: Supplementary file 2 [file Table_2.DOCX]

Intra-day and inter-day reproducibility values of the analytic batches.

| **FA** | **Intra-day variation** | | **Inter-day variation** | |
| --- | --- | --- | --- | --- |
|  | *mean ± d.s. (%)* | *CV (%)* | *mean ± d.s. (%)* | *CV (%)* |
| DHA  DPA  EPA  AA  Linolenic  Linoleic  Oleic  Stearic  Palmitic | 0.196±0.002  0.059±0.002  0.058±0.001  0.35±0.003  0.44±0.020  17.97±0.22  50.80±0.20  8.14±0.01  21.95±0.03 | 1.07  2.76  2.01  1.13  3.8  1.21  0.39  0.13  1.7 | 0.24±0.02  0.12±0.03  0.077±0.01  0.36±0.02  0.47±0.04  17.14±0.27  50.81±0.25  8.16±0.12  22.6±0.31 | 8.2  21.6  6.8  4.7  8.4  1.6  0.5  1.5  1.4 |

*FA= fatty acid; CV=coefficient of variation; DHA=docosahexaenoic acid; DPA= docosapentaenoic acid; EPA= eicosapentaenoic acid; AA= arachidonic acid.*
