# Supplementary material for: Maternal Supplementation With Krill Oil During Breastfeeding and Long-Chain Polyunsaturated Fatty Acids (LCPUFAs) Composition of Human Milk: A Feasibility Study
Source: Front Pediatr. 2018 Dec 20;6:407. doi: 10.3389/fped.2018.00407 (PMC6308297; doi:10.3389/fped.2018.00407)
Supplement: Supplementary file 3 [file Table_3.docx]

Supplementary Table 3. Characteristics of the infants in the two study groups and results of between-group comparison.

| **Infants’ characteristics** | **Group 1 (n=8)** | **Group 2 (n=8)** | **Group 1 vs. 2,**  **p-value** |
| --- | --- | --- | --- |
| Gestational age at birth (wks), median (IQR) | 35.3 (30.6-39.7) | 34.8 (31.5-39.8) | .798^°^ |
| Post-conceptional age at T0 (wks), median (IQR) | 40.3 (36.6-43.7) | 39.7 (37.3-43.8) | .798^°^ |
| No. of preterm neonates (GA ≤32 wks), n (%) | 4 (50%) | 4 (50%) | 1^§^ |

*^°^Mann-Whitney U test*

*^§^Fisher’s exact test*
